# High‐resolution crystal structures of the botulinum neurotoxin binding domains from subtypes A5 and A6

**DOI:** 10.1002/2211-5463.12931

**Published:** 2020-07-23

**Authors:** Jonathan R. Davies, Amy Britton, Sai Man Liu, K. Ravi Acharya

**Affiliations:** ^1^ Department of Biology and Biochemistry Claverton Down University of Bath Bath UK; ^2^ Department of Biochemistry and Biophysics Stockholm University Sweden; ^3^ Ipsen Bioinnovation Limited Milton Park Abingdon UK

**Keywords:** binding domain structure, botulinum neurotoxin, *Clostridium botulinum*, subtypes, X‐ray crystallography

## Abstract

*Clostridium botulinum* neurotoxins (BoNTs) cause flaccid paralysis through inhibition of acetylcholine release from motor neurons; however, at tiny doses, this property is exploited for use as a therapeutic. Each member of the BoNT family of proteins consists of three distinct domains: a binding domain that targets neuronal cell membranes (H_C_), a translocation domain (H_N_) and a catalytic domain (LC). Here, we present high‐resolution crystal structures of the binding domains of BoNT subtypes/A5 (H_C_/A5) and/A6 (H_C_/A6). These structures show that the core fold identified in other subtypes is maintained, but with subtle differences at the expected receptor‐binding sites.

## Graphical abstract

Botulinum neurotoxins (BoNTs) cause the deadly condition called botulism, but they can be used as therapeutics for a wide range of indications. BoNTs are classified into many different serotypes and subtypes, each with potentially different intoxication properties. Here, we report crystal structures of the receptor‐binding domains from two subtypes (BoNT/A5 and BoNT/A6) and compare their binding sites with previous BoNT/A structures.

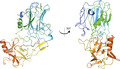

**Abbreviations**
BoNT: botulinum neurotoxin
GBS: ganglioside‐binding site
H_C_: cell‐binding domain
H_N_: translocation domain
LC: light chain

*Clostridial botulinum* neurotoxins (BoNTs) are responsible for causing the deadly condition, botulism, in vertebrates [1, 2, 3, 4]. There are seven distinct serotypes termed BoNT/A through BoNT/G, of which serotypes /A, /B, /E and /F^5^]. Each BoNT serotype can be further categorised into subtypes based on amino acid sequence identity. For example, there are currently eight known subtypes of BoNT/A (/A1‐/A8), which share between 84% and 97% sequence identity [6]. While BoNTs are the most toxic biological molecules known to science, they are used in human therapy, especially BoNT/A1 [7].

The BoNTs contain three major functional domains, a binding domain located in the C‐terminal half of the heavy chain (H_C_), a translocation domain located in the N‐terminal half of the heavy chain (H_N_) and a Zn^2+^‐dependent protease domain located in the light chain (LC). The H_C_ is responsible for targeting the BoNT to the neuronal cell membrane by binding to specific gangliosides and protein receptors on the neuronal cell surface. The H_N_ facilitates entry of the LC into the cytosol where it cleaves a target SNARE protein(s), which inhibits exocytosis. Although there are currently more than 46 different BoNT subtypes, there is limited structural information available for the majority of these natural variants. Many of these subtypes have been found to contain beneficial properties when compared to the commercially available toxins.

The BoNT subtypes from within the same serotype display a high degree of amino acid sequence identity and similarity; however, several studies have found distinct differences in their properties [8, 9, 10, 11, 12] (Fig. 1). Although the molecular basis of intoxication is not yet fully understood, the LC appears to define the length of intoxication (duration of action), while both H_N_ and H_C_ appear to be responsible for the spread and speed of cellular entry (onset of action). Considering the toxic nature of BoNTs, they are classed as tier 1 select agents due to their potential misuse in bioterrorism or as a bioweapon. From this perspective, structural details of each subtype may aid the design of broadly BoNT‐neutralising antibodies.

**Fig. 1:**
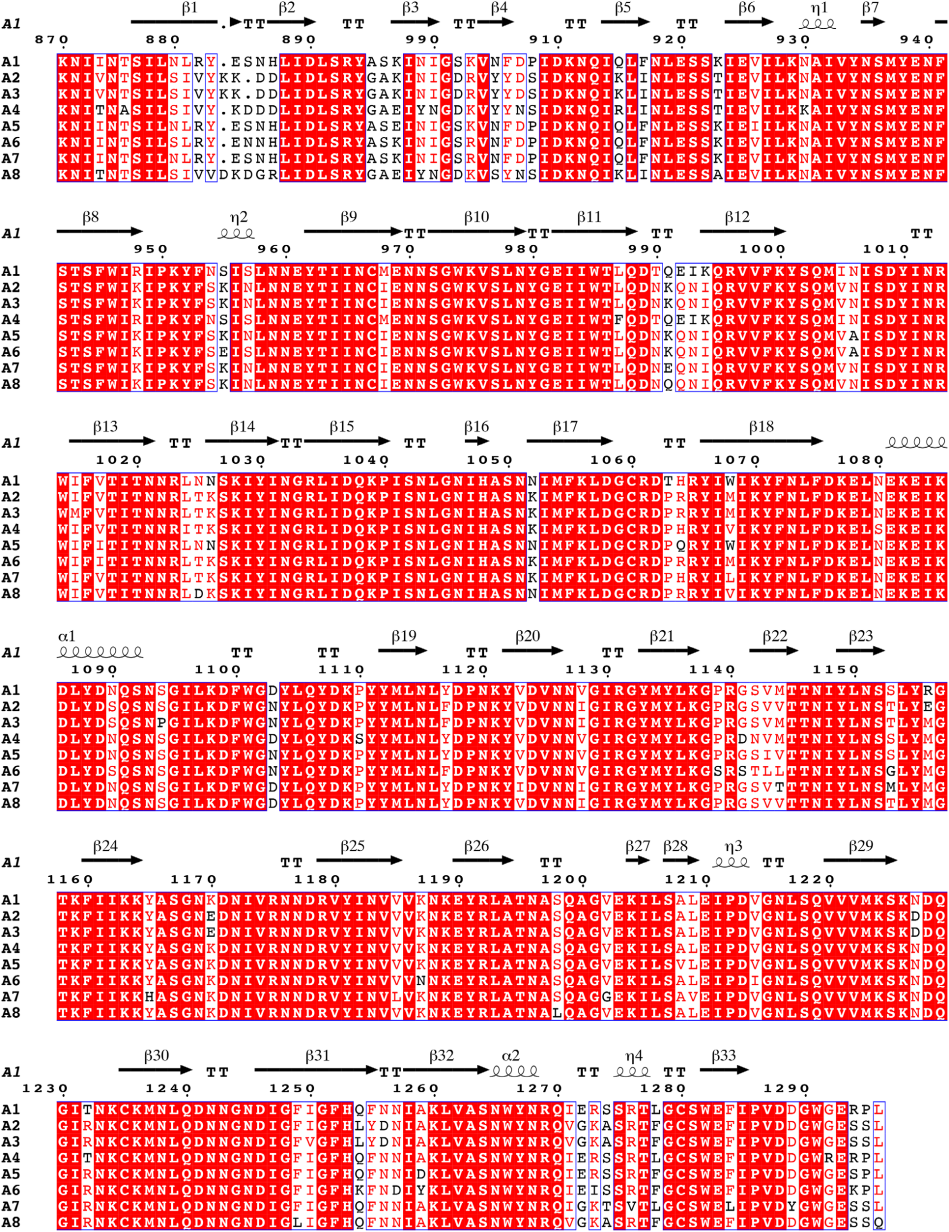
Alignment of the binding domain sequences from BoNT/A1 to A8. BoNT/A1 numbering and secondary structure used for annotation. Figure generated using ESPript [34]

Previously, we and others have determined the crystal structures of the binding domains from BoNT subtypes /A1, /A2, /A3 and /A4, and the related /HA alone [13, 14, 15, 16, 17], and in complex with various receptors: H_C_/A1‐GT1b [18], H_C_/A1‐SV2C [19, 20], H_C_/A2‐SV2C [14, 21] and H_C_/A3‐GD1a [22]. Here, we report the crystal structures of the BoNT/A5 and BoNT/A6 receptor‐binding domain and compare the binding sites with previous crystal structures of the BoNT/A subtype.

## Materials and methods

All reagents used were purchased from Sigma‐Aldrich (Dorset, UK) or Fisher Scientific (Leicestershire, UK) unless otherwise specified.

### Protein expression and purification

The binding domain (residues 871–1296) of BoNT/A5 and BoNT/A6 was cloned into the pJ401 vector (Atum Bio, California, USA) from their respective full‐length sequences (UniProtKB: C7BEA8 and C9WWY7) with an N‐terminal 6xHis tag. Constructs were expressed and purified as described previously [17]. The N‐terminal 6xHis tag was not removed from the proteins prior to crystallisation.

### Protein crystallisation

Crystallisation conditions were screened using commercially available 96‐well screens from Molecular Dimensions (Sheffield, UK) at 16 °C. H_C_/A5 (4 mg·mL^−1^) and H_C_/A6 (6 mg·mL^−1^) were dispensed using an Art Robbins Phoenix crystal screening nano‐dispenser into 96‐well 3‐drop Intelliwell plates (Molecular Dimensions, UK). Multiple screening kits from Molecular Dimensions were used. Crystals of H_C_/A5 were obtained using the sitting‐drop vapour diffusion method with 0.1 m sodium formate, 0.1 m ammonium acetate, 0.1 m sodium citrate tribasic dihydrate, 0.1 m sodium potassium tartrate tetrahydrate, 0.1 m sodium oxamate, 0.1 m imidazole, 0.1 m 2‐[N‐morpholino]ethanesulfonic acid, pH 6.5, 10%(v/v) ethylene glycol, 10%(w/v) PEG 8000 from (MORPHEUS screen, condition G2) and flash‐cooled in liquid nitrogen. Crystals of H_C_/A6, however, were obtained using the hanging‐drop vapour diffusion method and 0.2 m sodium acetate trihydrate, 0.1 m Bis‐Tris propane‐HCl pH 7.5, 22%(w/v) PEG 3350 (based on condition G7 of the PACT Premier screen) and flash‐cooled in liquid nitrogen after cryoprotection with 1 : 1 50%(v/v) glycerol in reservoir solution.

### X‐ray data collection and structure determination

Complete X‐ray diffraction data sets were collected from single crystals of H_C_/A5 and H_C_/A6 (3600 images each) using 0.1° oscillations and a wavelength of 0.98 Å at beamlines IO3 and IO4 (Diamond Light Source, Didcot, UK). Raw images were processed using DIALS [23], and integrated data were scaled and merged using Aimless [24] from the CCP4 suite [25]. The 3D structures of both proteins were solved by molecular replacement with PHASER [26] using the coordinates from Phyre2 web server homology models [27] as search models. Both models were manually built coot [28] and refined with refmac [29] in the CCP4 suite of programs [25]. The structures were validated with pdb_redo [30], molprobity [31] and wwpdb validation [32]. Crystallographic data processing and refinement statistics are given in Table 1. Structure‐based figures were generated with either PyMOL (Schrödinger, LLC, New York, NY, USA) or MOE (Chemical Computing Group, Quebec).

**Table 1:** X‐ray data collection and refinement parameters. Outer shell statistics are shown in parentheses

	H_C_/A5	H_C_/A6
Beamline	I03, DLS	I04‐1, DLS
Wavelength (Å)	0.9763	0.9159
Space group	P2_1_2_1_2_1_	P2_1_2_1_2_1_
Unit‐cell parameters		
a,b,c (Å)	43.55, 60.27, 185.15	39.54, 105.59, 112.41
α = β = γ (◦)	90, 90, 90	90, 90, 90
Resolution (Å)	92.57–1.15 (1.17–1.15)	112.41–1.35 (1.37–1.35)
*R*_merge_ (%)	0.087 (1.098)	0.094 (1.725)
*R*_meas_ (%)	0.090 (1.158)	0.099 (1.839)
*R*_pim_ (%)	0.026 (0.363)	0.030 (0.631)
CC_1/2_ (%)	0.998 (0.447)	0.999 (0.463)
Mean < I/σ(I)>	11.1 (1.9)	10.7 (0.9)
Completeness (%)	100 (99.5)	100 (99.7)
No. of observed reflections	2,107,443 (84,519)	1,099,418 (43,053)
No. of unique reflections	173,797 (8,450)	104,434 (5,129)
Multiplicity	12.1 (10.0)	10.5 (8.4)
Refinement statistics		
*R*_work_/*R*_free_	0.135/0.161	0.147/0.168
RMSD bond lengths (Å)	0.02	0.01
RMSD bond angles (◦)	2.20	1.66
Ramachandran statistics (%)		
Favoured	96.3	97.0
Allowed	3.7	3.0
Outliers	0	0
Wilson B‐factor (Å^2^)	11.4	13.5
Average B‐factors (Å^2^)		
Protein	16.8	20.3
Water	32.6	31.5
No. of atoms		
Protein	3827	3633
Water	522	423
PDB code	6TWP	6TWO

## Results and Discussion

### Structure of the BoNT/A5‐binding domain (H_C_/A5)

**Fig. 2:**
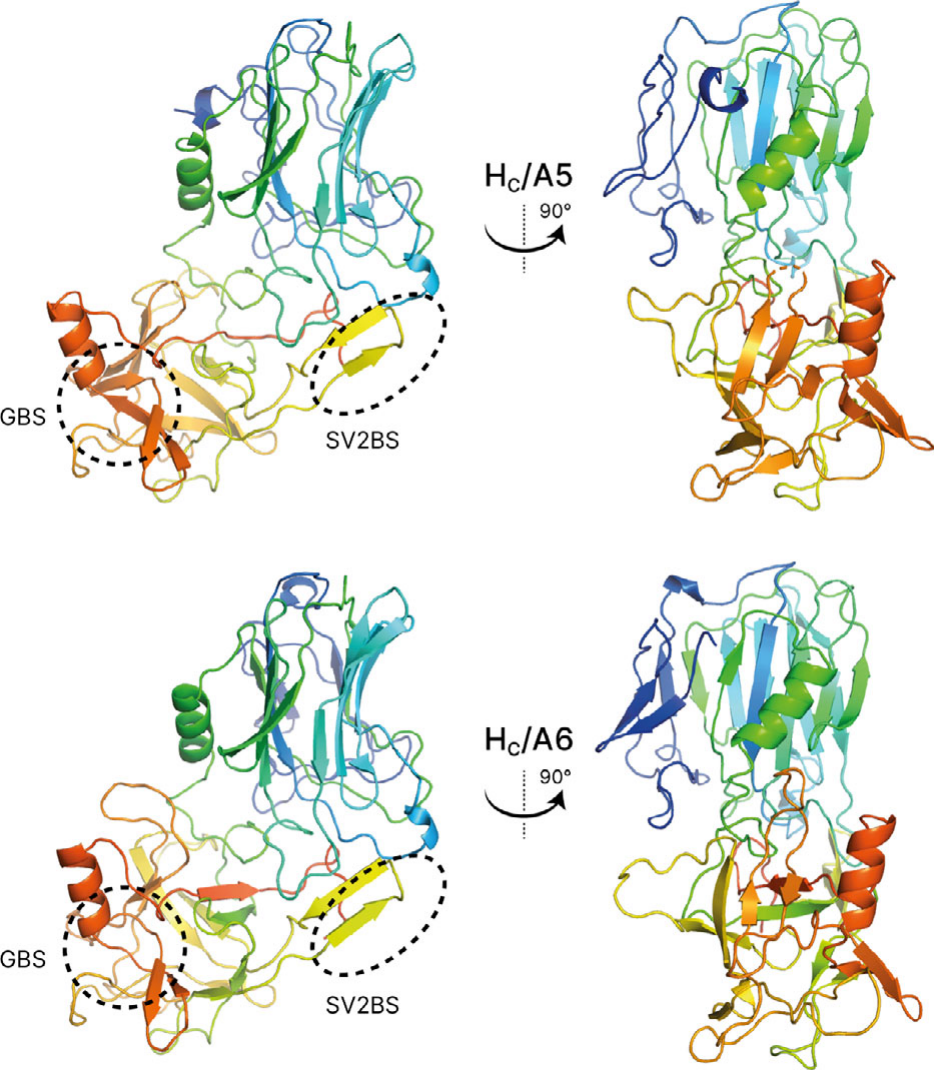
Crystal structures of H_C_/A5 and H_C_/A6. Overall structure of the binding domain (H_C_) of BoNT/A5 (top) and BoNT/A6 (bottom). Putative ganglioside‐ and SV2‐binding sites are indicated by a dashed ellipse labelled GBS and SV2BS, respectively.

**Fig. 3:**
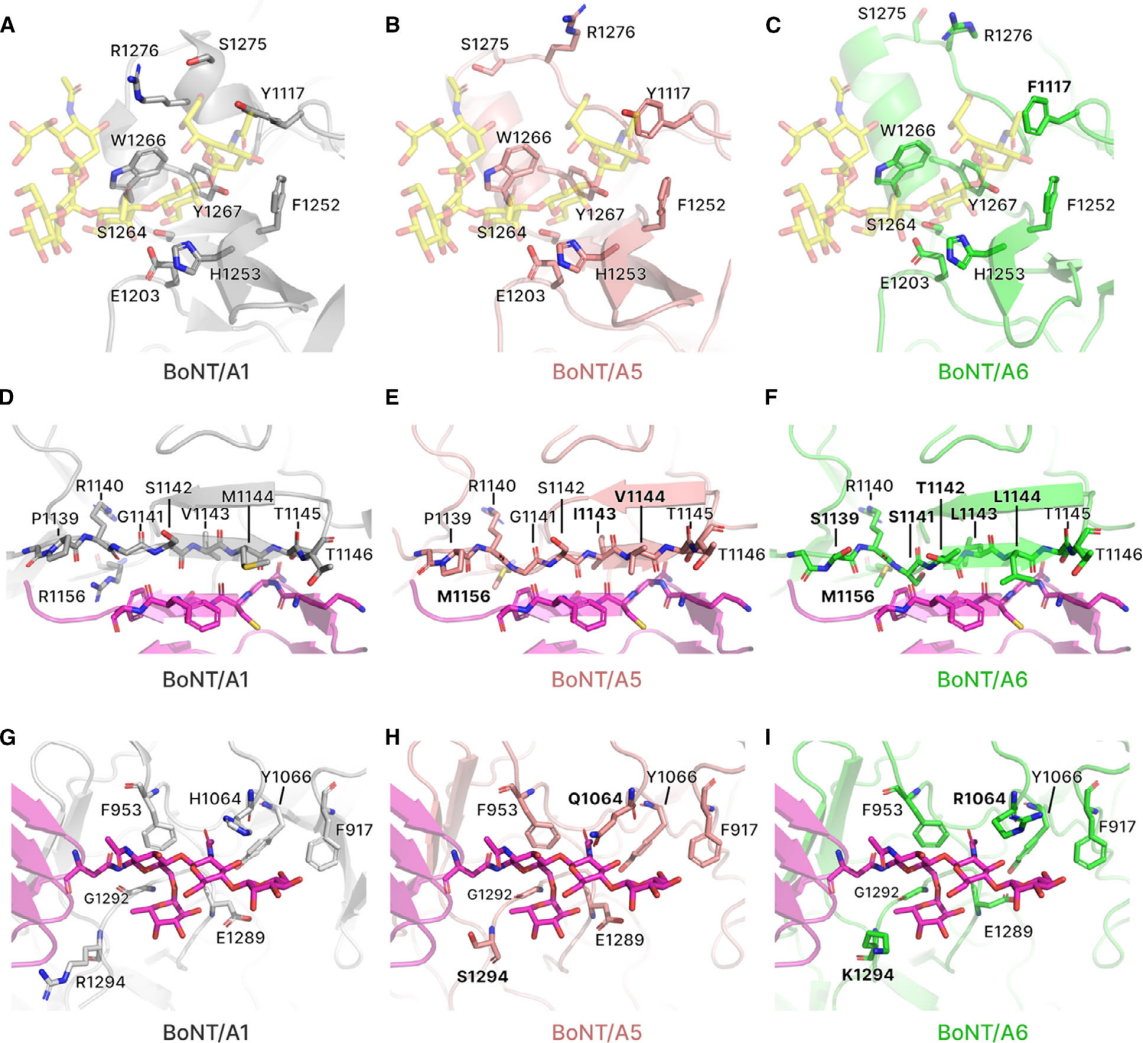
Comparison of receptor‐binding sites of BoNT/A5 and BoNT/A6. (A‐C) Comparison of the ganglioside‐binding site of H_C_/A1 (PDB: 2VU9), H_C_/A5 and H_C_/A6. Note that the ganglioside, GT1b (yellow), has been superposed into the putative site of H_C_/A5 and H_C_/A6. (D‐F) Comparison of the SV2‐binding site of H_C_/A1 (PDB: 5JLV), H_C_/A5 and H_C_/A6. Note that the protein receptor, SV2C (purple), has been superposed into the putative site of H_C_/A5 and H_C_/A6. (G–I) Comparison of the SV2 glycan‐binding site of H_C_/A1 (PDB: 5JLV), H_C_/A5 and H_C_/A6. Note that the SV2C glycans (purple) have been superposed into the putative site of H_C_/A5 and H_C_/A6. Residues involved in receptor binding are indicated and those which differ from BoNT/A1 are highlighted in bold.

**Fig. 4:**
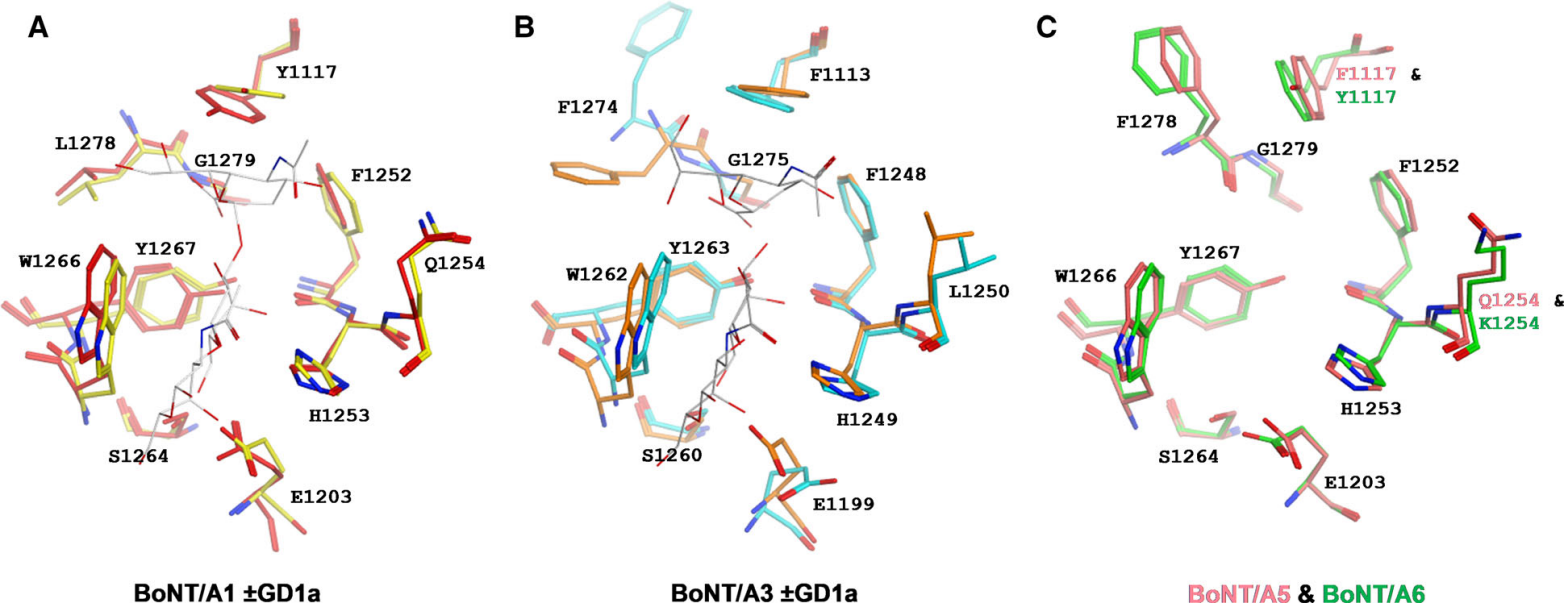
Comparison of the GD1a‐binding site. (A) The GD1a‐binding site of BoNT/A1 is shown in the bound (red; PDB: 5TPB) and unbound (yellow; PDB: 3BTA) conformation (the interacting sugar moieties are shown in white). (B) The GD1a‐binding site of BoNT/A3 is shown in the bound (orange; PDB: 6THY) and unbound (cyan; PDB: 6F0O) conformation (the interacting sugar moieties are shown in grey). (C) The putative GD1a‐binding site of BoNT/A5 (pink) and BoNT/A6 (green) is shown in the unbound conformation. Note that residues at positions 1117, 1254 and 1278 either match the corresponding residue in BoNT/A1, BoNT/A3 or neither.

The crystal of H_C_/A5 belonged to the orthorhombic space group *P*2_1_2_1_2_1_, and it diffracted to a resolution of 1.15 Å (Table 1). Electron density was excellent throughout, with all H_C_/A5 residues (except the N‐terminal 6xHis tag and Lys871) being easily observed. The structure closely resembles the structures of other BoNT‐binding domains [6] with an N‐terminal jelly roll‐like fold and C‐terminal modified β‐trefoil fold containing a conserved ganglioside‐binding site (SxWY) (Fig. 2). However, compared to the structure of BoNT/A1 in complex with GT1b (PDB: 2VU9), the loop of residues 1260–1280, which contains ganglioside‐interacting residues, adopts a different arrangement (Fig. 3a,b). It is possible that upon ganglioside binding, the loop changes conformation to allow S1275 and R1276 to take part in the interaction. In comparison with the unbound GD1a‐binding site of BoNT/A1 (PDB: 3BTA) and BoNT/A3 (PDB: 6F0O), the corresponding site in H_C_/A5 perhaps more resembles that of the latter rather than the former, which is consistent with a higher sequence identity between the sites at residues corresponding to positions 1117, 1254 and 1278 (Figs 1 and 4). Either way, considering that both BoNT/A1 and BoNT/A3 are able to bind to GD1a (PDB: 5TPB and 6THY, respectively), this suggests that BoNT/A5 is able to do so too.

**Fig. 5:**
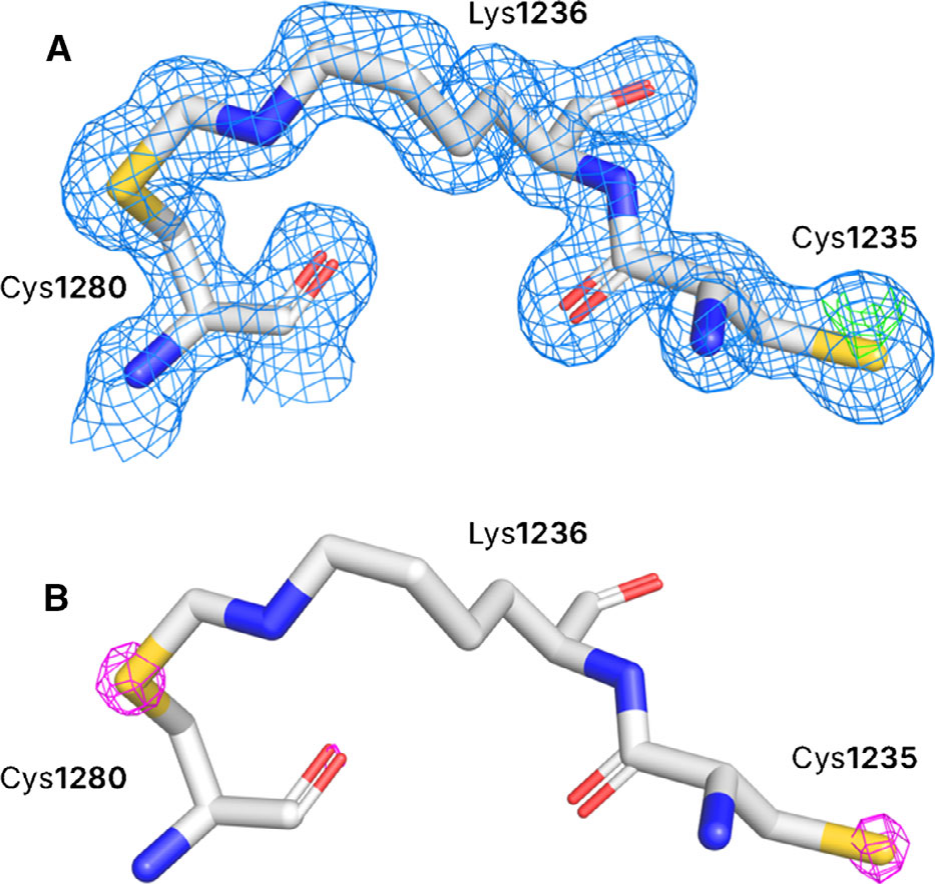
Electron density around Cys1280 and Lys1236 in H_C_/A5. (A) The electron density around Cys1280 and Lys1236 is clear and continuous, indicating the presence of a covalent bond between the two side chains. The 2mFo‐Fc map (blue) is contoured to 0.5 e/Å^3^, and the Fo‐Fc (negative: red; positive: green) map is contoured to 0.46 e/Å^3^. (B) A weak sulfur‐SAD signal within the diffraction data enabled the calculation of an anomalous map (contoured to 0.06 e/Å^3^; magenta), which indicates the precise position of each sulfur atom for each Cys residue.

In close proximity to the ganglioside binding loop was observed an unusual feature – a methylene bridge between the S_γ_ of Cys1280 and N_ζ_ of Lys1236 (Fig. 5a), rather than a disulfide bond with a nearby cysteine residue (Cys1235). During refinement of the H_C_/A5 structure, clear electron density was observed between the side chains of Cys1280 and Lys1236, into which a methylene group could be fitted. Weak anomalous data recorded at the start of data collection were used to generate a low‐resolution anomalous difference map. Despite the noise, large peaks were observed at the location of sulfur atoms, which confirmed the location of each cysteine residue (Fig. 5b). This specific methylene bond between a lysine and cysteine side chain is unusual, and the mechanism surrounding the formation of a methylene‐bridged lysine and cysteine is not fully understood [33]. Whether this bond is biologically relevant remains to be established. While there are indications of this bond in the electron density maps of other BoNT crystal structures, it is possible that this may be an artefact of exposure to synchrotron radiation.

Inspection of the H_C_/A5 structure corresponding to the BoNT/A1 SV2C‐binding site (^1139^PRGSVMTT^1146^ + Arg1156) reveals the presence of perhaps a slightly shortened β‐hairpin (Fig. 3d,e). The three different residues at positions 1143, 1144 and 1156 (V → I, M → V and R → M, respectively) do not appear to preclude the possibility of SV2C binding. Indeed, the related binding domain of BoNT/HA possesses the same residues at the corresponding location and is still able to bind to SV2C [20]. However, inspection of the accompanying SV2C glycan‐binding site reveals one residue (Gln1064) potentially hindering the binding of glycan (Fig. 3g,h). This residue, corresponding to His1064 in BoNT/A1, has been shown to drastically decrease the binding affinity to SV2C [20]. Although this suggests that SV2C may not be the protein receptor for BoNT/A5, it should be noted that there exists a second BoNT/A5 sequence that differs by this one residue (UniProtKB: C1IPK2).

### Structure of the BoNT/A6‐binding domain (H_C_/A6)

The crystals of H_C_/A6 belong to orthorhombic space group *P*2_1_2_1_2_1_ and diffracted to a resolution of 1.35 Å. Electron density was excellent, with all but the first six residues of H_C_/A6 being clearly observed, and like H_C_/A5, the overall protein fold was highly similar to other BoNT H_C_ structures. The ganglioside‐binding site was identical to that of H_C_/A5 except for residue 1117, which was a Phe rather than a Tyr (Fig. 3b,c). Although the absence of the hydroxyl group would result in the loss of hydrogen bonding with the terminal sialic acid of GT1b, the side chain can still continue to interact with the carbon ring. Compared to the unbound GD1a‐binding site of BoNT/A1 (PDB: 3BTA) and BoNT/A3 (PDB: 6F0O), the corresponding site in H_C_/A6 also more resembles that latter rather than the former, even though there is no greater sequence identity between the sites at residues corresponding to positions 1117, 1254 and 1278 (Figs 1 and 4). Like that for the H_C_/A5 structure, BoNT/A6 is predicted to be able to bind to GD1a as well.

For the corresponding BoNT/A1‐SV2C‐binding site in H_C_/A6, a larger sequence variation is observed: ^1139^SRSTLLTT^1146^ + Met1156 rather than ^1139^PRGSVMTT^1146^ + Arg1156 for BoNT/A1. Despite these differences, the β‐hairpin remains available to bind to SV2C via mostly backbone–backbone hydrogen bonding (Fig. 3d,f). Like H_C_/A5, H_C_/A6 possesses a different residue in the glycan‐binding site at position 1064 (Arg) compared to that of BoNT/A1 (His, Fig. 3g,i), and this has also been reported to significantly reduce binding of glycosylated SV2C [20]. This would suggest that BoNT/A6 may have a lower affinity for SV2C than BoNT/A1. Interestingly, BoNT/A2 also contains an Arg at position 1064 and it has previously been reported that both BoNT/A2 and BoNT/A6 are capable of entering hiPSC‐derived neurons faster than BoNT/A1 [8].

## Conclusions

The BoNT/A subtypes are believed to bind to the target cell surface via a dual‐receptor complex involving a ganglioside and protein receptor. For BoNT/A1, they are GT1b (preferentially) and SV2C, respectively, but for most of the others, the exact identities of these receptors have not yet been determined. Structural analysis of the expected binding sites has revealed some differences with that of BoNT/A1, suggesting either an altered binding affinity to each receptor or a different receptor specificity altogether. Our high‐resolution structures further add to the body of knowledge around BoNT receptor binding and enhance the available molecular information for engineering novel therapeutic BoNTs and BoNT‐binding moieties.

## Author contributions

JRD performed purification and crystallisation of H_C_/A5, supervised AB, processed and analysed the data, and drafted the manuscript. AB performed purification and crystallisation of H_C_/A6. SML analysed the data and edited the manuscript. KRA supervised the study, analysed the data, and edited the manuscript.

## Conflict of interest

The authors JRD, AB and KRA from the University of Bath declare no competing financial interests. SML is an employee of Ipsen Bioinnovation Limited.

## Data Availability

Accession codes: The atomic coordinates and structure factors (codes 6TWP and 6TWO) have been deposited in the Protein Data Bank (http://www.pdb.org).
